# Supplementary figures and images for: Metabolomic profiling of pancreatic adenocarcinoma reveals key features driving clinical outcome and drug resistance
Source: eBioMedicine. 2021 Apr 13;66:103332. doi: 10.1016/j.ebiom.2021.103332 (PMC8054161; doi:10.1016/j.ebiom.2021.103332)

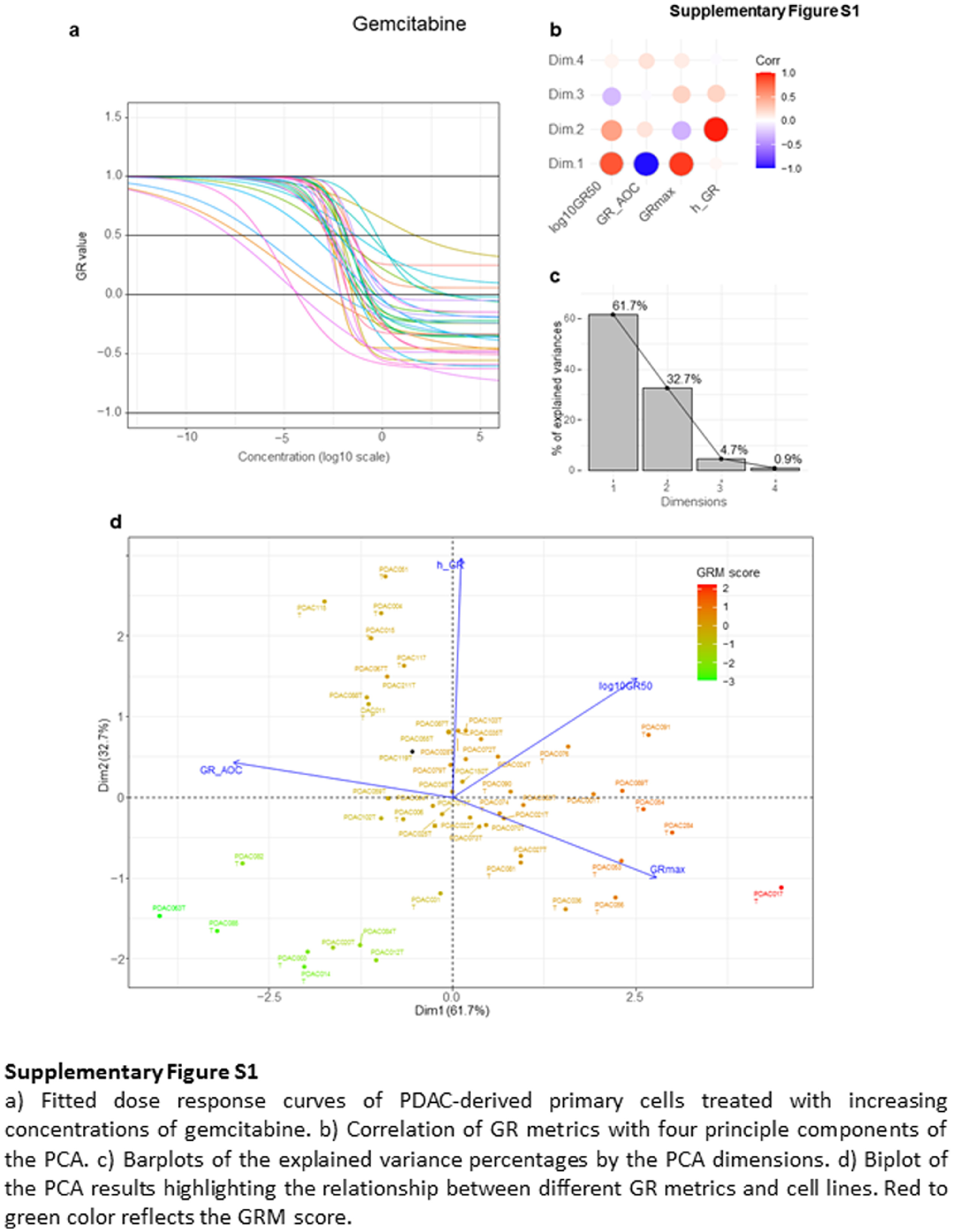

Supplement: Supplementary file 5 [file mmc5.jpg]

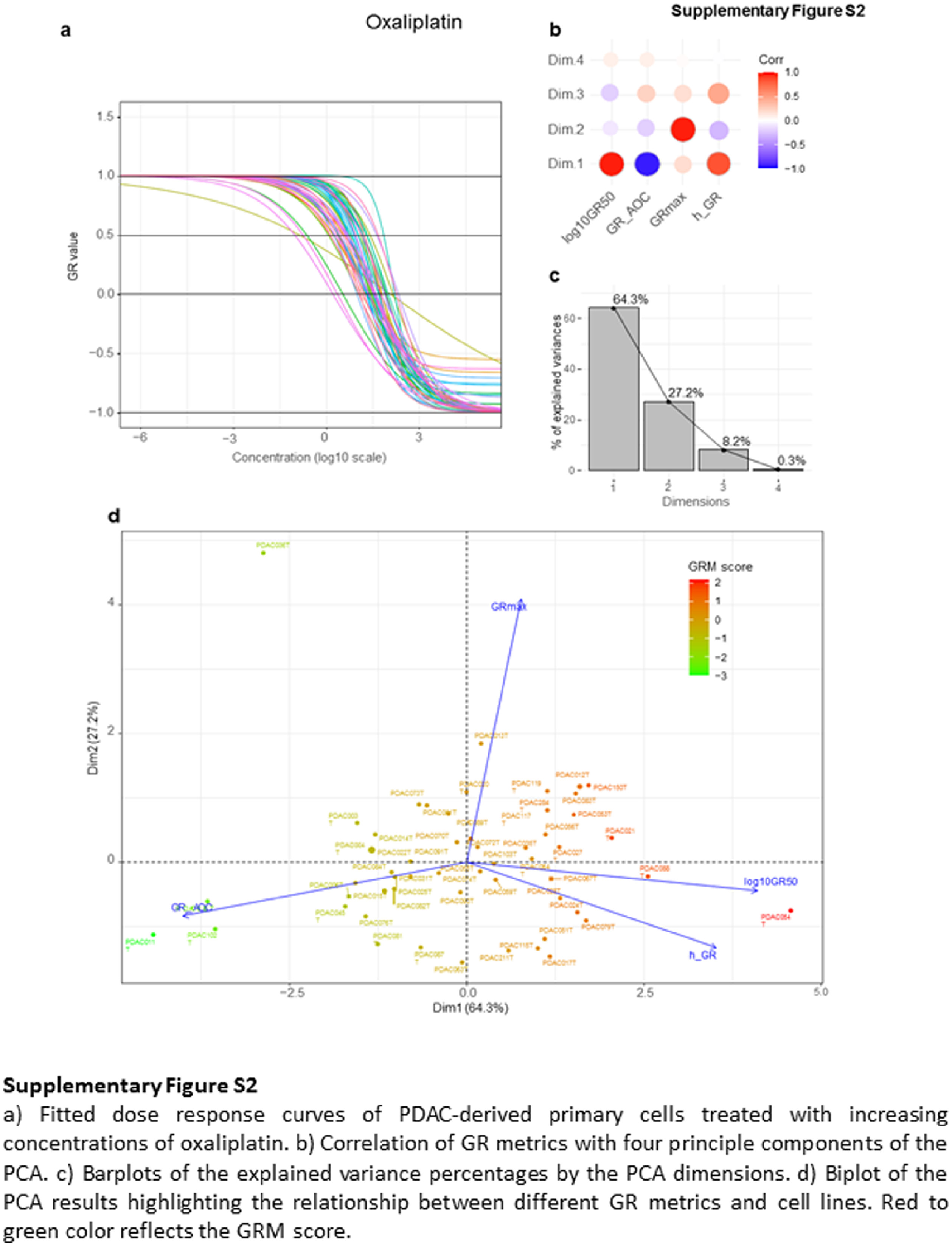

Supplement: Supplementary file 6 [file mmc6.jpg]

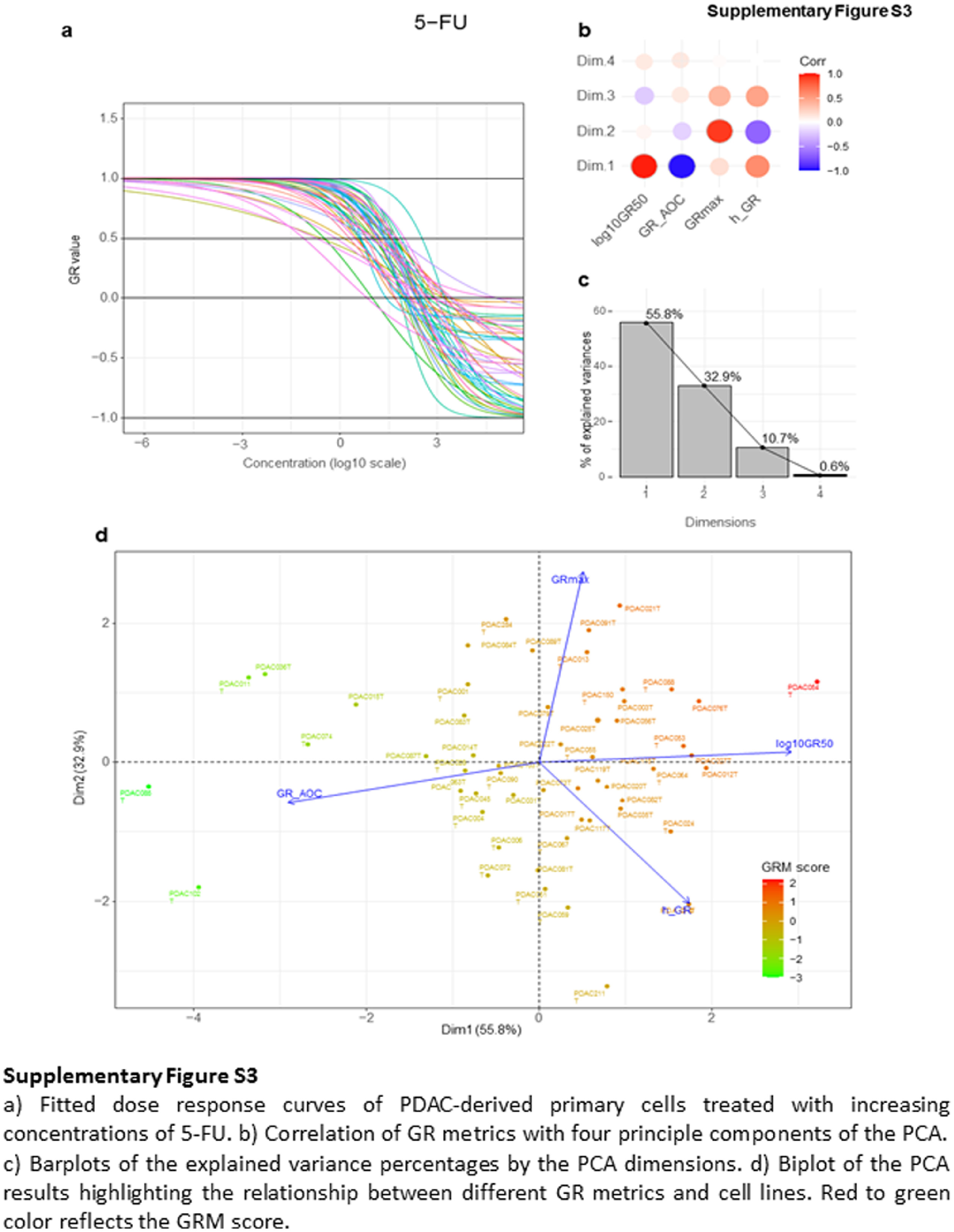

Supplement: Supplementary file 7 [file mmc7.jpg]

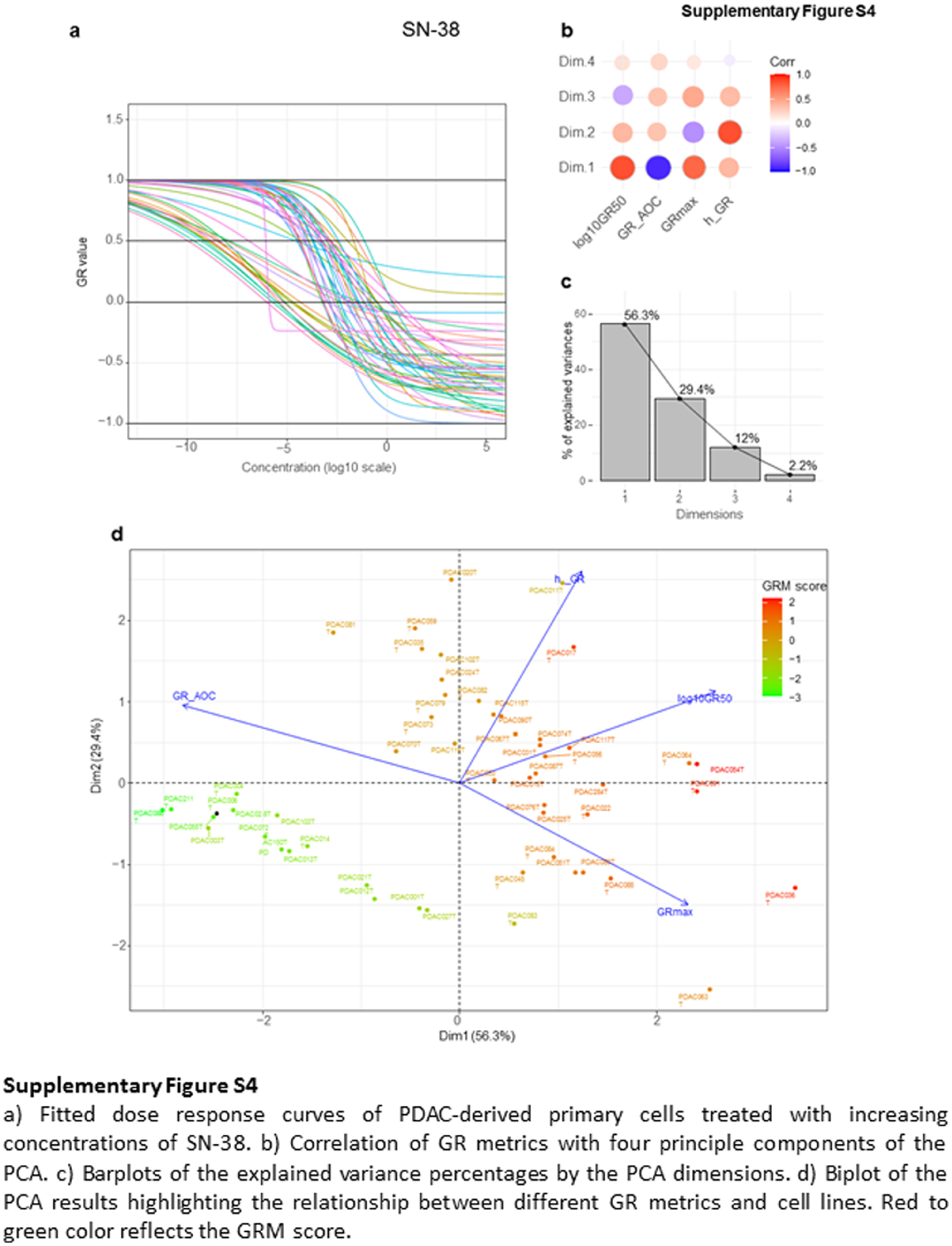

Supplement: Supplementary file 8 [file mmc8.jpg]

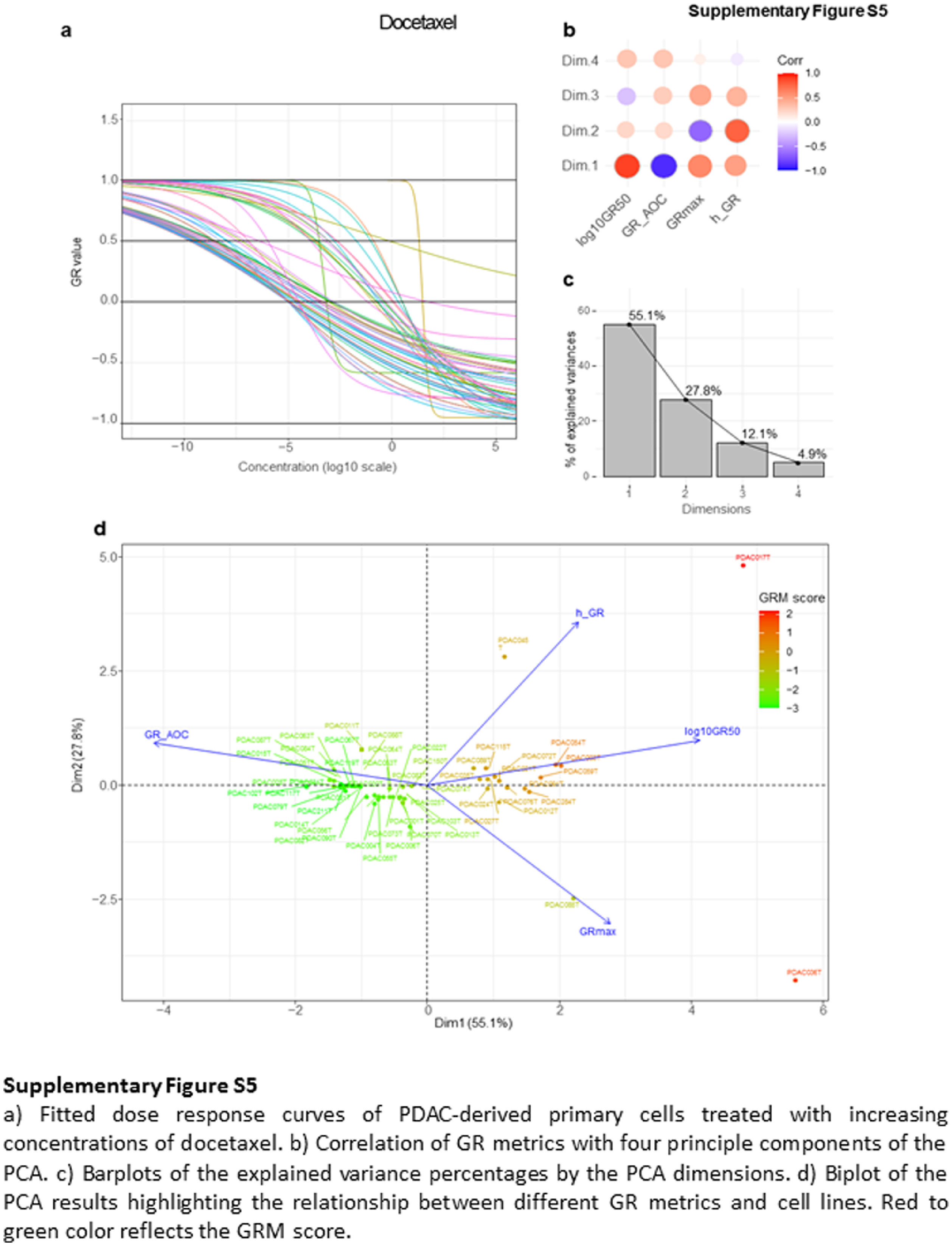

Supplement: Supplementary file 9 [file mmc9.jpg]

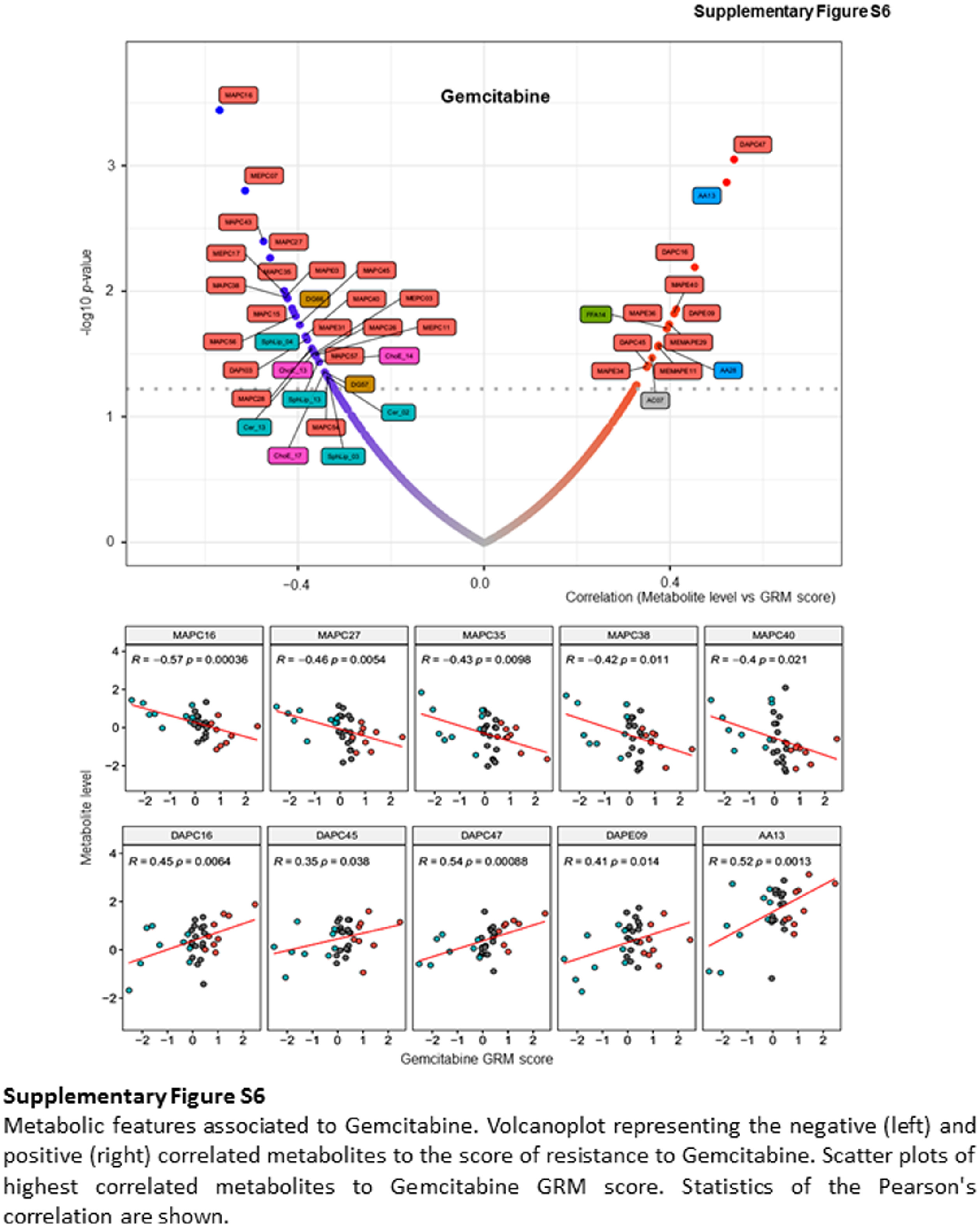

Supplement: Supplementary file 10 [file mmc10.jpg]

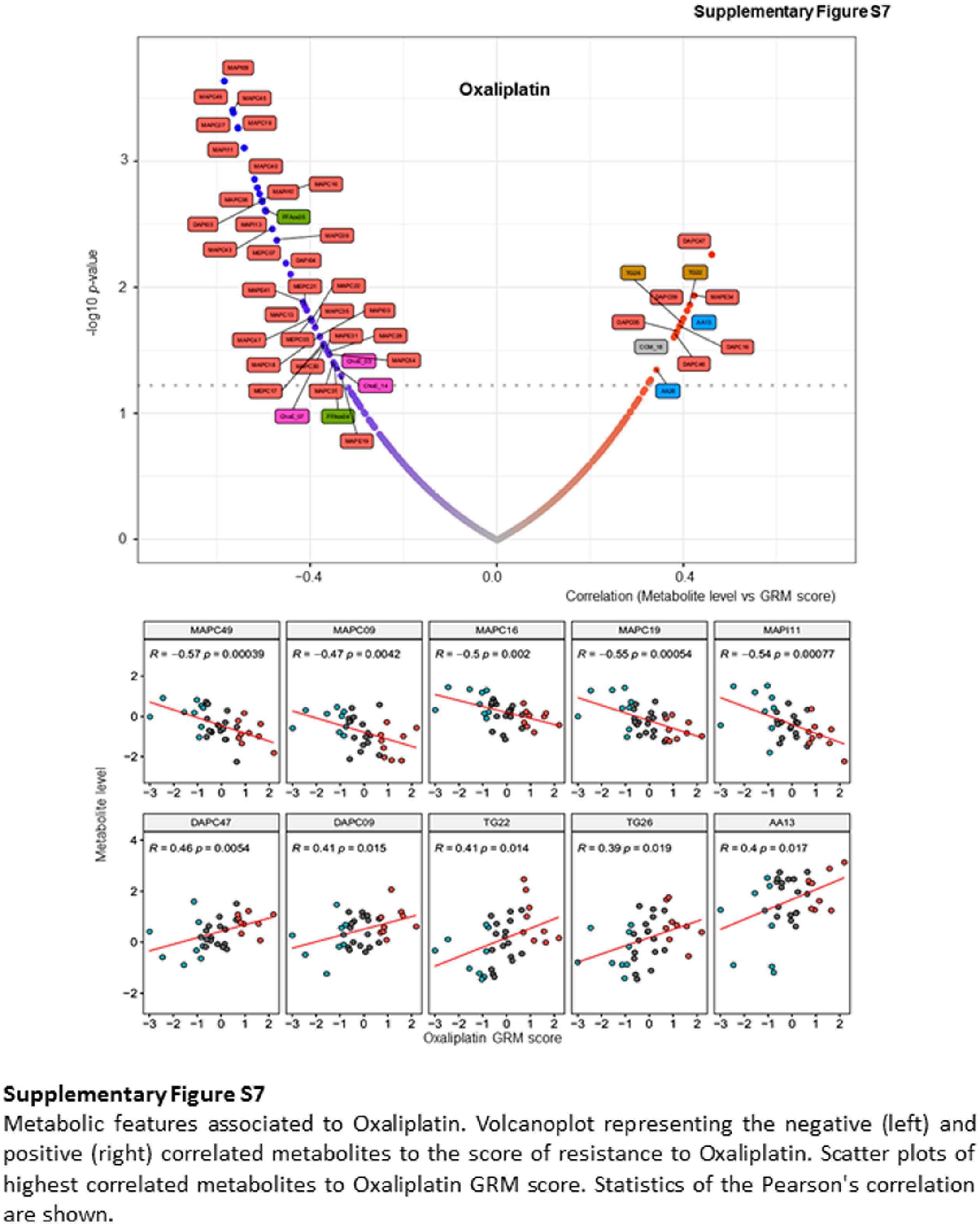

Supplement: Supplementary file 11 [file mmc11.jpg]

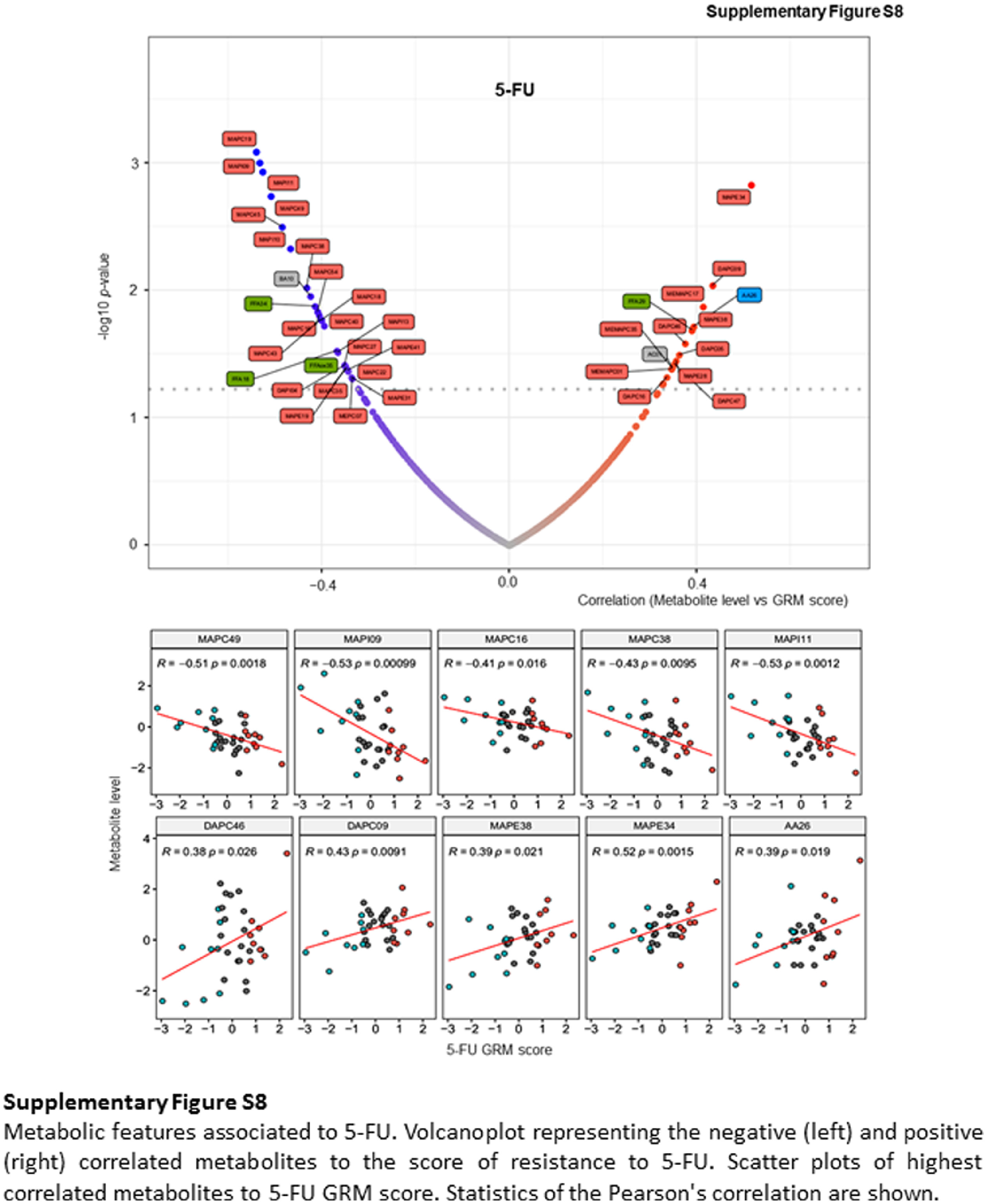

Supplement: Supplementary file 12 [file mmc12.jpg]

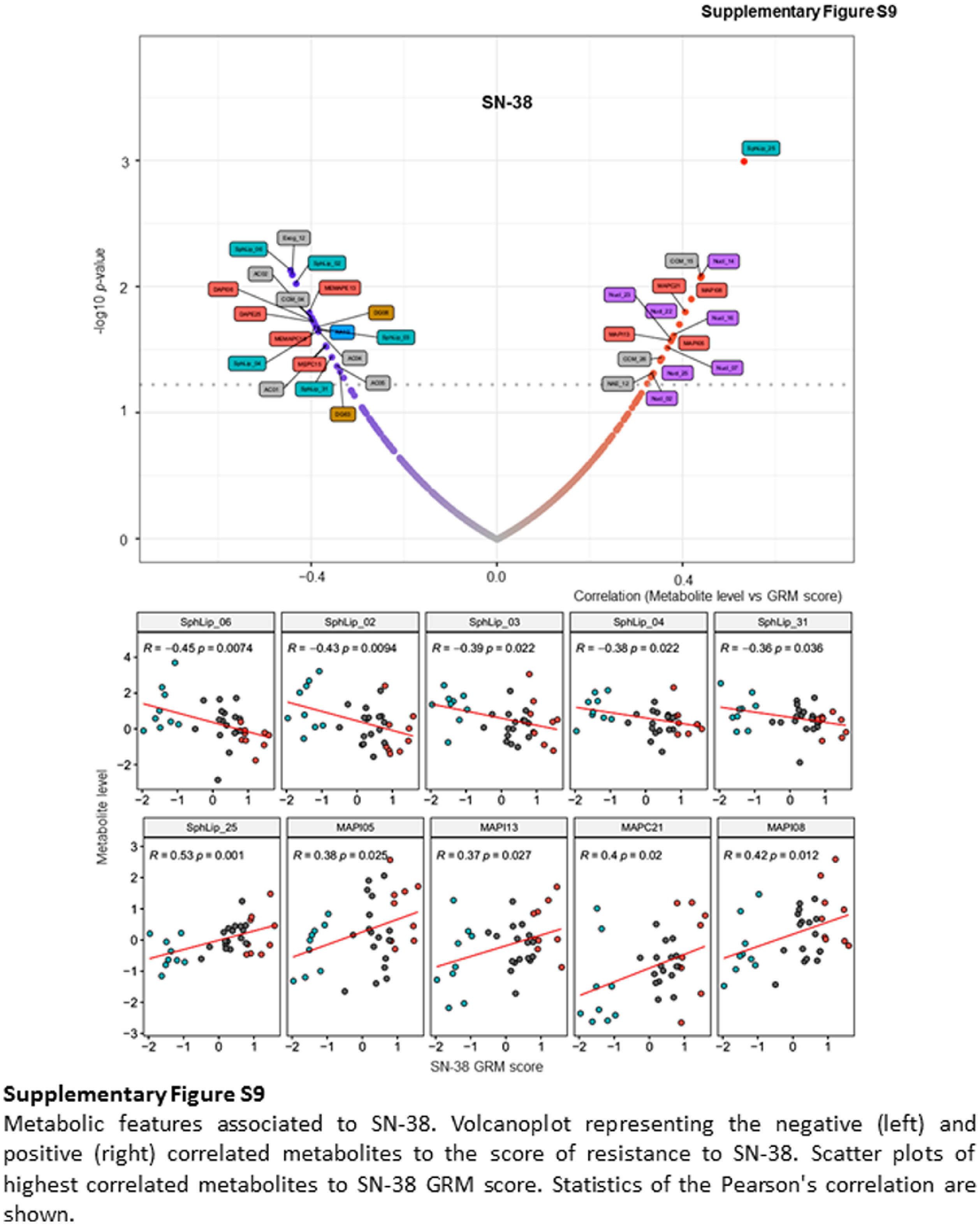

Supplement: Supplementary file 13 [file mmc13.jpg]

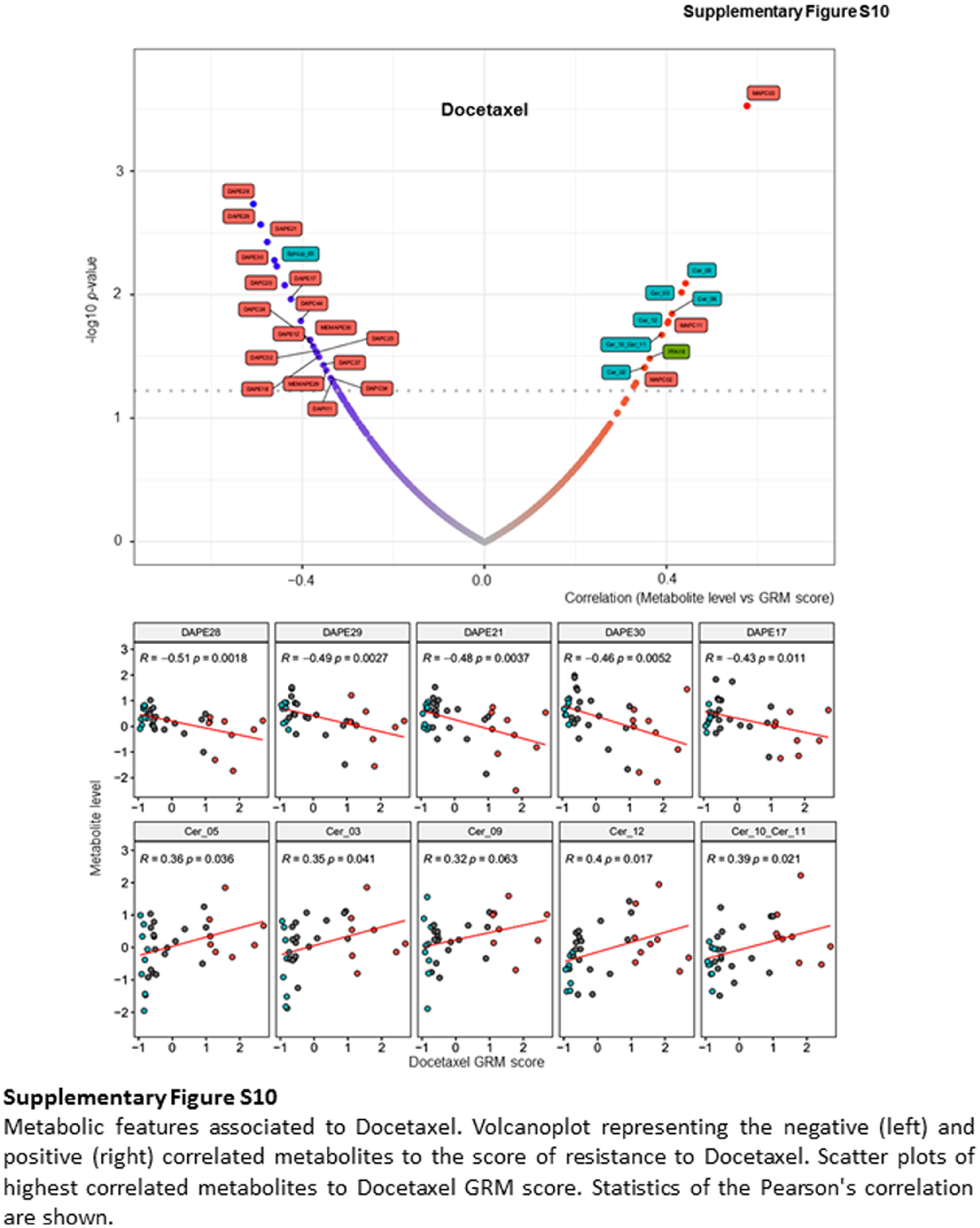

Supplement: Supplementary file 14 [file mmc14.jpg]

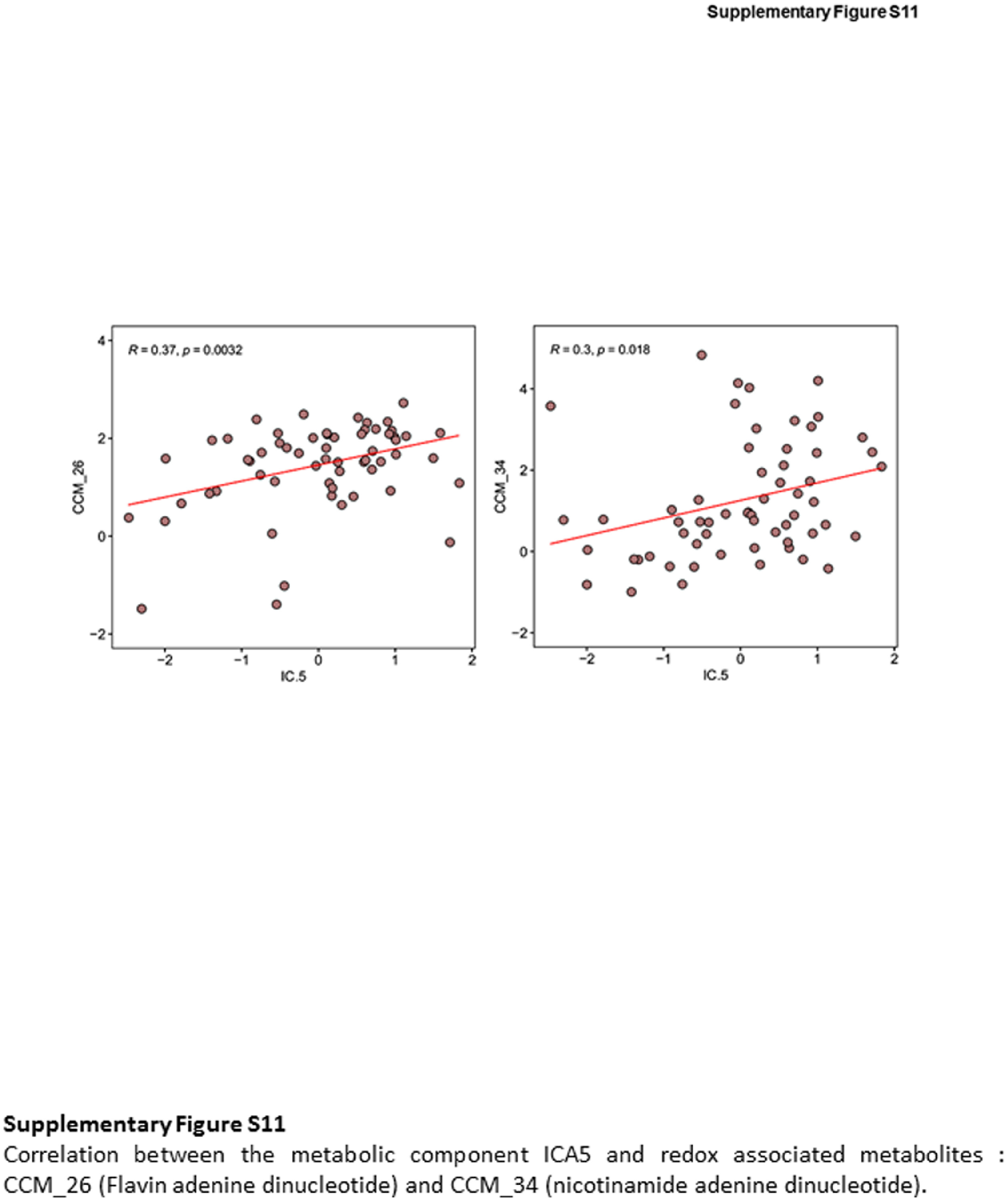

Supplement: Supplementary file 15 [file mmc15.jpg]

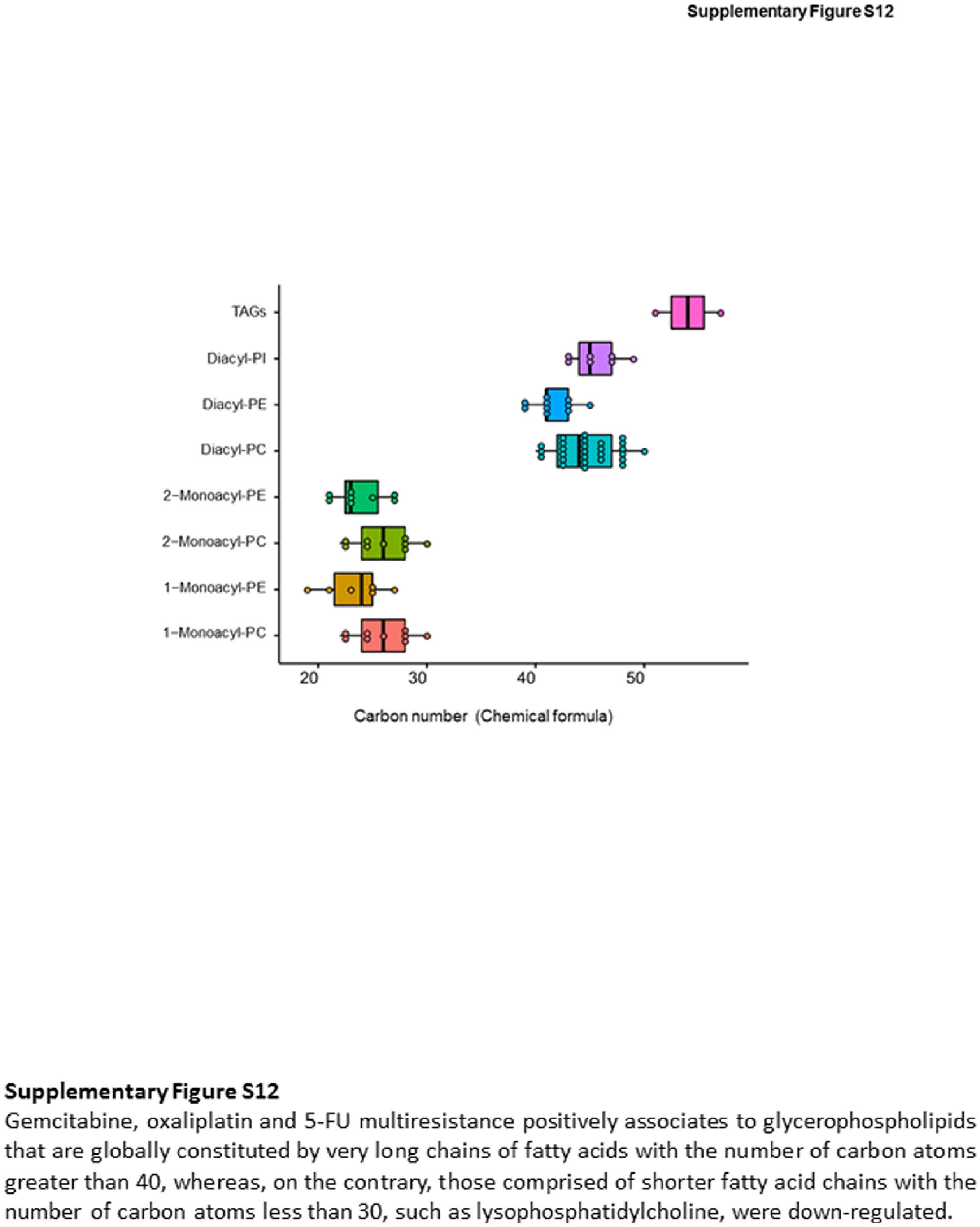

Supplement: Supplementary file 16 [file mmc16.jpg]
